# Ulnar-sided pain due to extensor carpi ulnaris tendon subluxation: a case report

**DOI:** 10.1186/1752-1947-6-394

**Published:** 2012-11-22

**Authors:** Hakan Cift, Korhan Ozkan, Salih Söylemez, Feyza Unlu Ozkan, Hacer Burcu Cift

**Affiliations:** 1 Department of Orthopaedics and Traumatology, Sb. Medeniyet University Goztepe Research and Training Hospital, Istanbul, Turkey; 2 Department of Physical Therapy and Rehabilitation, Fatih Sultan Mehmet Education and Research Hospital, Istanbul, Turkey; 3 Department of Anesthesia and Reanimation, Fatih Sultan Mehmet Education and Research Hospital, Istanbul, Turkey; 4 Bostanci mah. Mehmet Sevki Pasa cd. Isik apt. 32/12, Istanbul, Turkey

**Keywords:** Extensor carpi ulnaris, Tendon subluxation

## Abstract

**Introduction:**

We present the case of a patient with extensor carpi ulnaris tendon subluxation who was first treated for distal radioulnar joint sprain.

**Case presentation:**

A 25-year-old Caucasian man was seen at our policlinic one month after he had fallen on his outstretched hand. A diagnosis of extensor carpi ulnaris subluxation was made clinically but we also had the magnetic resonance imaging scan of the patient’s wrist which displayed an increased signal on T2-weighted images consistent with inflammation around the extensor carpi ulnaris tendon. The extensor carpi ulnaris tendon was found to be dislocating during supination and relocating during pronation. The sheath was reconstructed using extensor retinaculum due to attenuation of subsheath.

**Conclusion:**

There was no recurrent dislocation of the extensor carpi ulnaris tendon of the patient at his last follow up 12 months after the operation.

## Introduction

The extensor carpi ulnaris (ECU) is a muscle located in the human forearm that acts to extend and adduct the wrist. It crosses through the sixth dorsal compartment where it is held tightly to the ulnar groove by a subsheath. Traumatic recurrent dislocations of the ECU tendon are rare injuries caused by tearing of the subsheath of the wrist, particularly on forearm pronation and supination. Tenderness and swelling are often present over the ECU tendon at the ulnar head [1].

In this report we aim to present the case of a patient with ECU tendon subluxation who was first treated for distal radioulnar joint sprain.

## Case presentation

**Figure 1:**
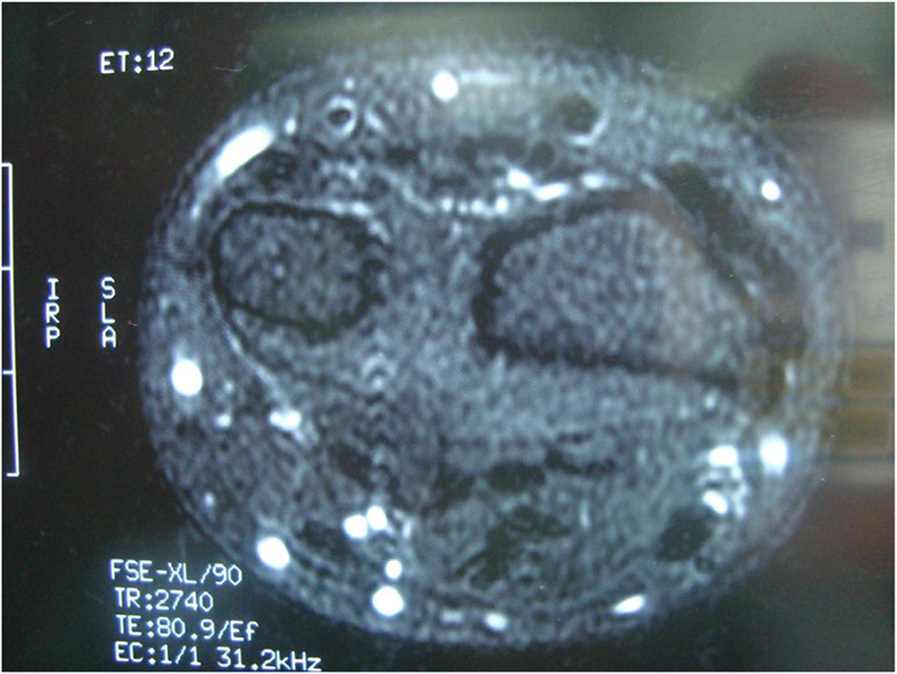
T2 magnetic resonance images; extensor carpi ulnaris tendon with inflammation around sheath.

A 25-year-old Caucasian man was seen at our policlinic one month after he had fallen on his outstretched hand. He was first treated with a below arm cast with the wrist at neutral flexion for a distal radioulnar joint sprain. Four weeks later his cast was removed and we found that he was still having pain and swelling of the ECU tendon during pronation and supination of the forearm. Although the ECU subluxation diagnosis was made clinically we also had the MRI (magnetic resonance imaging) scan of the patient’s wrist which displayed an increased signal on T2-weighted images consistent with inflammation around the ECU tendon (Figure 1).

**Figure 2:**
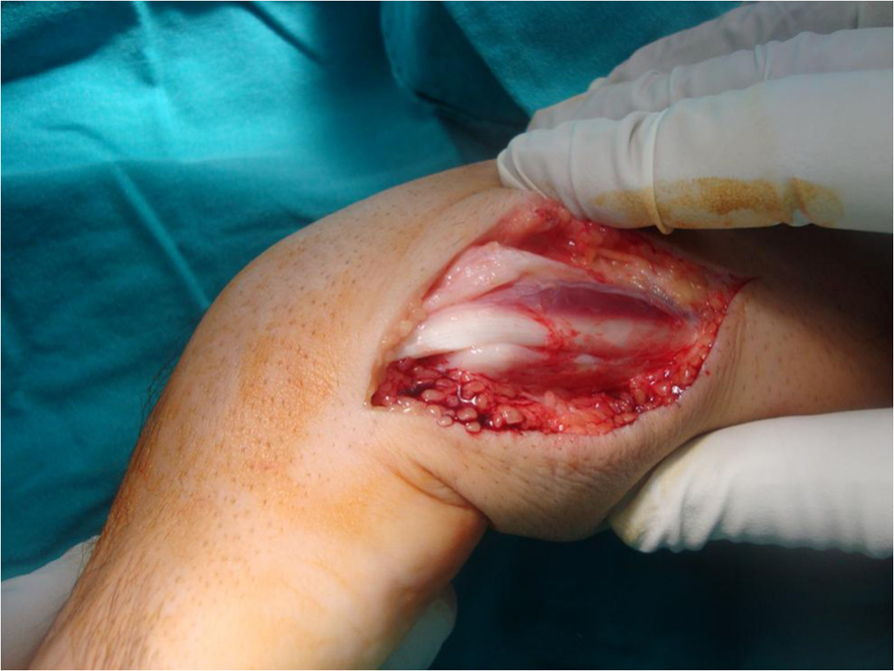
Extensor carpi ulnaris tendon seems in the groove with torn subsheath.

**Figure 3:**
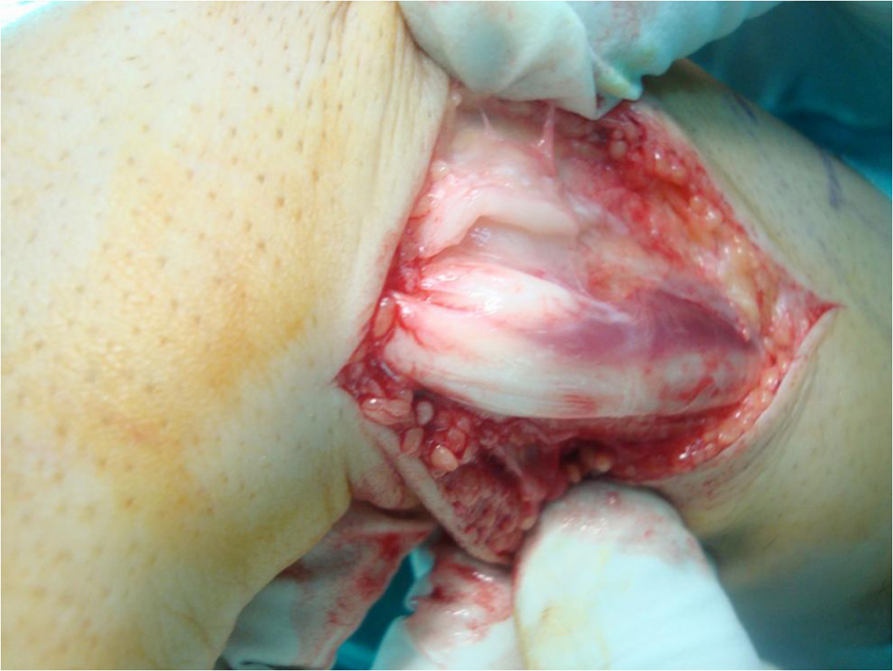
Extensor carpi ulnaris tendon dislocated from groove by supination of the wrist.

Surgical exploration was carried out using axillary block anesthesia under pneumatic tourniquet control. The patient’s extensor retinaculum was found to be intact which was opened longitudinally. However, his fibrosseus sheath which was tightly binding the tendon at the ulnar groove was found to be torn from the ulnar attachment (Figure 2). The ECU tendon was found to be dislocating during supination (Figure 3) and relocating during pronation.

The sheath was reconstructed using extensor retinaculum due to attenuation of the subsheath.

After the operation the patient’s arm was kept in a long arm cast for five weeks with the elbow in 90º of flexion and neutral forearm rotation. The reason for keeping his arm in a long arm cast for five weeks was to prevent relocation. The patient returned to normal activities four months after the operation.

**Figure 4:**
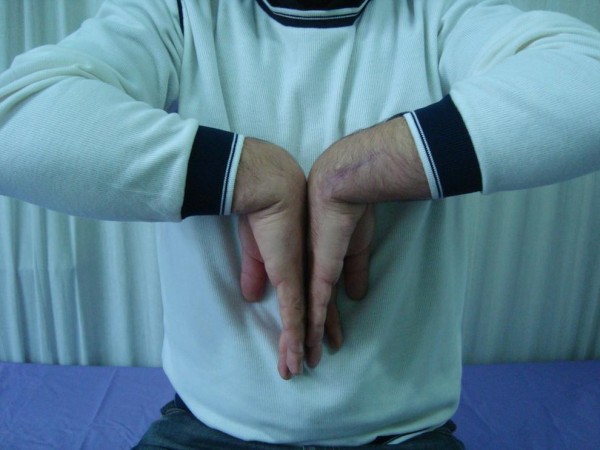
12 months after operation with full wrist flexion.

**Figure 5:**
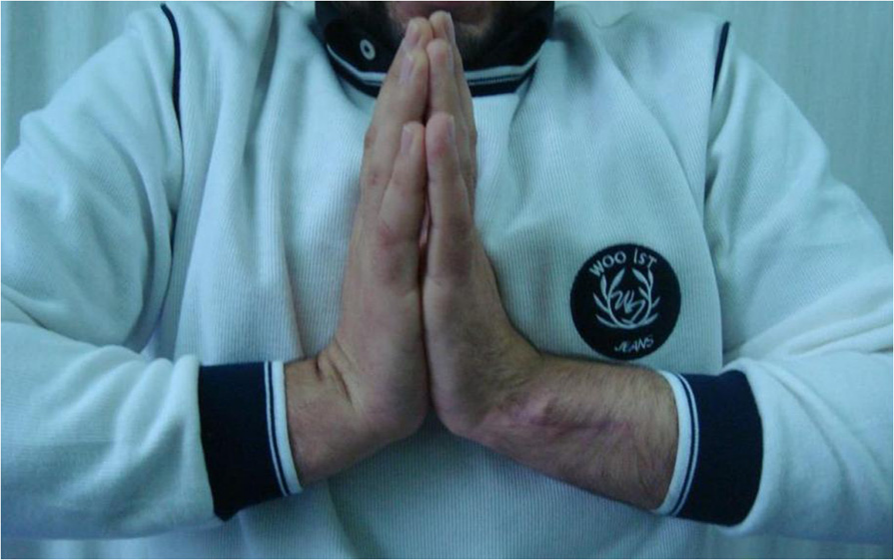
12 months after operation with full wrist extension.

There was no recurrent dislocation of the ECU tendon of the patient at his last follow up 12 months after the operation (Figures 4 and 5).

## Discussion

The mechanism causing ECU subluxation is often forced supination, palmar flexion and ulnar deviation of the wrist. The ECU dislocates during supination and relocates with pronation. ECU instability was first described by Vulpius in 1964 [2]. Spinner and Kaplan described the tension of the tendon of ECU in its intact fibrosseus tunnel as an important stabilizing factor for the distal radioulnar joint. The stability of the joint during supination is maintained by the tendon being retained in its groove [3].

There are usually two types of disruption of the fibrosseus sheath: a tear may occur on the ulnar side as in our case, or disruption may occur on the radial side.

In acute subluxation, immobilization for six weeks in a long arm cast with the forearm pronated and the wrist in a slight radial deviation and dosiflexion may be done, but in chronic and symptomatic subluxation, surgical reconstruction of the subsheath should be considered [4]. According to Rowland, surgical treatment of ECU luxation may be considered even in an acute case due to the inadequate potential for anatomic healing of the fibrosseus sheath [5].

## Conclusion

Tension of the tendon of the ECU in its intact fibrosseus tunnel is an important stabilizing factor for the distal radioulnar joint.

As in our case, undefined pain of the ulnar wrist often poses a diagnostic challenge and ECU tendon dislocation should be suspected in differential diagnosis [6-8]. On supination the tendon displaces, often with an audible snap, and on pronation it relocates to its normal place in the ulnar groove.

Although we achieved satisfactory results with surgical treatment, more study is needed if this pathology is to be treated with conservative methods accurately in acute cases.

## Consent

Written informed consent was obtained from the patient for publication of this manuscript and accompanying images. A copy of the written consent is available for review by the Editor-in-Chief of this journal.

## Competing interests

The authors declare that they have no competing interests.

## Authors’ contribution

SS, HBC and FUO contributed to the conception and design of the study, carried out the literature research, manuscript preparation and manuscript review. HC was involved with the case and writing of the manuscript and the general management of the patient. KO revised the manuscript for important intellectual content. All authors read and approved the final manuscript.
